# Non-Hodgkin lymphoma after pediatric kidney transplantation

**DOI:** 10.1007/s00467-021-05205-6

**Published:** 2021-10-11

**Authors:** Ryszard Grenda

**Affiliations:** Department of Nephrology, Kidney Transplantation & Hypertension, Children’s Memorial Health Institute, Warsaw, Poland

**Keywords:** Non-Hodgkin lymphoma (NHL), Kidney transplantation, Risk factors, Management

## Abstract

Non-Hodgkin lymphoma (NHL) that develops after kidney transplantation belongs to post-transplant lymphoproliferative disorders (PTLD) occurring with an incidence of 2–3%. Most pediatric cases are related to primary infection with Epstein-Barr virus (EBV), able to transform and immortalize B cells and widely proliferate due to the lack of relevant control of cytotoxic T cells in patients receiving post-transplant immunosuppression. NHL may develop as a systemic disease or as a localized lesion. The clinical pattern is variable, from non-symptomatic to fulminating disease. Young age of transplant recipient, seronegative EBV status at transplantation, and EBV mismatch between donor and recipient (D+/R-) are regarded as risk factors. Immunosuppression impacts the development of both early and late NHLs. Specific surveillance protocols, including monitoring of EBV viral load, are used in patients at risk; however, detailed histopathology diagnosis and evaluation of malignancy staging is crucial for therapeutic decisions. Minimizing of immunosuppression is a primary management, followed by the use of rituximab in B-cell NHLs. Specific chemotherapeutic protocols, adjusted to lymphoma classification and staging, are used in advanced NHLs. Radiotherapy and/or surgical removal of malignant lesions is limited to the most severe cases. Outcome is variable, depending on risk factors and timing of diagnosis, however is positive in pediatric patients in terms of graft function and patient survival. Kidney re-transplantation is possible in survivors who lost the primary graft due to chronic rejection, however may be performed after at least 2–3 years of waiting time, careful verification of malignancy-free status, and gaining immunity against EBV.

## Introduction

Every lymphoid malignancy developing after organ transplantation is per definition classified as a post-transplant lymphoproliferative disorder (PTLD). Non-Hodgkin lymphoma (NHL) is one of several subtypes of this wide spectrum of conditions; therefore, relevant data strictly related to this malignancy are “hidden” among other information, available in databases and registries combining data of pediatric and adult patients and different solid organ transplantations (SOT) and covering all types of PTLD. All PTLDs meeting the histopathological criteria of classical NHL are evaluated according to the histopathological classification system of the World Health Organization (WHO 2017; Table 1) which distinguishes four major types of PTLD, with two sub-categories, varying from early non-destructive (plasmacytic hyperplasia, infectious mononucleosis, florid follicular hyperplasia) to destructive polymorphic, monomorphic (B-cell, T-cell, NK-cell further classified according to the lymphoma they resemble in the immunocompetent host), and (more rarely) classical Hodgkin lymphoma. In practice, the direct differentiation between separate WHO categories is not always possible without additional investigations, mainly related to several aspects of EBV infection [1]. Monomorphic NHLs account for 35–83% of all PTLD cases in children who underwent SOT, and diffuse large B-cell lymphomas (DLBCL) represent the most frequent single entity (>30%) [2].

**Table 1.** Types of post-transplant lymphoproliferative disorders (PTLD) in the revised 2017 WHO (World Health Organization) histopathological classification system [1].

**Non-destructive PTLDs** Plasmatic hyperplasia Infectious mononucleosis Florid follicular hyperplasia **Polymorphic PTLD** **Monomorphic PTLDs **(classified according to the lymphoma they resemble) B-cell neoplasms Diffuse large B-cell lymphoma Burkitt lymphoma Plasma cell myeloma Plasmacytoma Other T-cell neoplasms Peripheral T-cell lymphoma, NOS Hepatosplenic T-cell lymphoma Other **Classic Hodgkin lymphoma PTLD**	

## Incidence

The incidence of post-transplant NHL (among other PTLDs) is specific to the type of transplanted organ. The data vary between different databases, settings, and transplantation eras, and the relevant numbers are limited to the whole group of PTLD. The overall 10-year incidence of PTLD in adult SOT recipients reported by UNOS (United Network for Organ Sharing) was 0.7% in kidney, 1% in liver, 1% in heart and pancreas, and 2% in lung transplantation [3]. The highest incidence of PTLD (19%) was reported in intestinal and multiorgan transplantation, which could be translated to almost 20 times higher relative risk compared with kidney transplantation (239.5 vs. 12.6) [4]. The variable organ-specific incidence of PTLD was also reported in children (20% in intestinal, 15% in lung, 5–10% in liver, 6% in heart, and 2–3% in kidney transplantation) [2, 5].

Comparison of cancer incidence between the transplanted (*n* = 951, including 400 kidneys) and non-transplanted pediatric population (*n* = 5.3 mln) in Ontario, Canada, showed that the risk of PTLD/lymphoma is 128.4 times higher and the event rate/1000 patient-years is 7.8 (vs. 0.1) [6]. The annual incidence (per million inhabitants) in the USA (according to data from US National Cancer Institute’s Surveillance, Epidemiology and End Results Program) steadily increases with age from 5.9 in children <5 years of age, 10 in children between 5 and 10 years, to 15 in adolescents (compared to 150 in adults) [7]. In the population of SOT recipients, the risk of developing NHL is much higher (up to >200 times); therefore, more than a half of post-transplant malignancies are lymphomas [8–10]. Data from 16 US Cancer Registries participating in the Transplant Cancer Match Study (1990–2012) included a total of 279 pediatric SOT recipients (46.7% of them were kidney transplants) with a confirmed NHL diagnosis. The majority (64.5%) of the cases were DLBCL. The distribution of age at diagnosis was 23.7% for the age of 0–4, 19.7% for 5–9, 33% for 10–14, and 23.7% for 15–19 years. The incidence of NHL in SOT recipients was 257 times higher than in the general age-matched population (306 vs. 1.19 per 100.00 person-years). While in the general population the incidence of NHL increased with age, in the transplant population, it was the highest in young children (< 5 years of age). As the kidney was the most frequently transplanted solid organ in this registry, renal graft recipients constituted the majority of DLBCL cases in the whole registry [11]. Overall, 74% of PTLD cases in pediatric kidney transplant recipients in the Austrian registry presented the poly- or monomorphic form of NHL [12]. In general, PTLD occurs more commonly (especially in pediatric patients) as an EBV-positive (>90%), early (<12 months after transplantation) than as a late event (>1 year post-transplant). However, there is a biphasic pattern of PTLD with a second peak which occurs between 7 and 10 years post-transplant. About half of late cases are EBV-negative and present monomorphic B-cell pattern [13, 14]. A report by the German multicenter pediatric PTLD registry, including 52 kidney transplant recipients (among a total of 127 SOT patients) showed that most (83.3%) of early PTLDs were B-cell NHLs and their incidence was lower in the late-onset PTLD subgroup (63.5%; *p* = 0.024) [15].

## Presentation features of NHL in pediatric transplant recipients

PTLD should be suspected in patients with fever accompanied by sore throat, tonsillar enlargement with exudates, and cervical lymphadenopathy. The lesions may be localized in the mandibula, jaw bone, hard or soft palate, buccal mucosa, gingiva, and lips. Some patients may present gastrointestinal symptoms, including anorexia, nausea, vomiting, abdominal pain, and bleeding, This may be suggestive of mucocutaneous ulceration, intestinal presence of mass lesions, or bowel perforation. Hepatosplenomegaly may also be present in some cases.

## Clinical staging

In general, PTLD may develop as a systemic disease or as a localized lesion. At early stages, it represents indolent, EBV-positive polyclonal lymphoproliferation, while advanced NHL (or other type of lymphoma) occurring in a later period is a more aggressive disease of variable localization. Defining the clinical staging of each individual case is important from that point, and the International Pediatric Non-Hodgkin Lymphoma Staging System [16] serves as a diagnostic tool (Table 2). Specificity of primary site involvement was analyzed in a group of 82 pediatric solid organ recipients, including 11 patients after kidney transplantation, with a mean time from transplantation to diagnosis of 1.44 years (0.15–4.15). B-cell-positive and early PTLD cases predominated. The most frequent localization was tonsillar/adenoidal (T/A) localization (34%), followed by gastrointestinal (32%), miscellaneous (central nervous system, kidney, lung, and soft tissue; a total of 12%), lymph node (11%), and multisite pattern (11%). In general, T/A localization was related to better patient (PTLD-related) survival compared with other sites (*p* = 0.012) [17]. Other reports indicate significant effect of the nervous system localization (PCNSL) on post-transplant mortality (stage IV). An extended US study, including a total of 299,029 recipients of solid organs (7.3% of all were pediatric cases; 58% patients were after kidney transplantation), revealed that the overall risk of PCNSL was higher than in systemic NHL (IR 65.1 and 11.5, respectively; both compared to the general population) and the overall risk was higher in kidney recipients than liver recipients (adjusted incidence rate ratio; aIRRin liver (vs. kidney) was 0.52) and higher in patients receiving alemtuzumab (aIRR = 3.12) and polyclonal antibodies (aIRR = 2.03) and being EBV-seronegative at the time of transplantation (aIRR 1.95). Most cases occurred within the first 1.5 years after the transplantation, with high mortality (aIRR 11.79, as compared to transplant patients with no malignancy, and 4.80, as compared with systemic NHL). The overall incidence of PCNSL in the pediatric subgroup was lower than in adult patients (aIRR = 0.83) [18]. Data from the German Pediatric PTLD registry showed that the presence of stage IV lymphomas (of CNS or bone marrow localization) was an independent risk factor for poor patient survival (*p* = 0.0005), while a non-significant difference in survival was seen between early- and late-onset, mono- or polymorphic, and EBV-seropositive and EBV-negative PTLD cases [19]. A recent report from the European Intergroup for Childhood NHL showed data of a cohort of CNS-localized lymphomas in 25 transplanted children, including 11 kidney recipients, of whom 4 developed polymorphic PTLDs, 7 DLBCL, 2 mature B-cell NHL NOS, and one developed T-cell lymphoblastic lymphoma. The overall 4-year survival rate for the group was 100% for monomorphic and 69±11% for polymorphic malignancies [20].

**Table 2.** The International Pediatric Non-Hodgkin Lymphoma Staging System [16].

Stage 1 Single tumor with exclusion of mediastinum and abdomen (N, BM, EN, N, S)
Stage II Single EN tumor with regional node involvement ≥ Two N areas on the same side of the diaphragm Primary GI tract tumor (usually in the ileocecal area) ± involvement of associated mesenteric nodes that is completely resectable (if there is malignant ascites or extension of the tumor to adjacent organs, it should be regarded as stage III)
Stage III ≥ Two EN tumors (including EN-B or EN-S) above and/or below the diaphragm ≥Two N areas above or below the diaphragm Any intrathoracic tumor (mediastinal, hilar, pulmonary, pleural, or thymic) Intra-abdominal and retroperitoneal disease, including liver, spleen, kidney, and/or ovary localizations, regardless of the degree of resection (except primary GI tract tumor, usually in the ileocoecal region) ± involvement of associated mesenteric nodes that is completely resectable Any paraspinal or epidural tumor, regardless of whether other sites are involved Single B lesion with concomitant involvement of EN and/or nonregional N sites
Stage IV Any of the above findings with initial involvement of CNS (stage IV CNS), BM (stage IV BM), or both (stage IV combined) based on conventional methods

*N* nodal, *B* bone, *BM* bone marrow, *EN* extranodal, *S* skin, *CNS* central nervous system

## Risk factors

There are several risk factors of PTLD/NHL identified in patients after organ transplantation, based on registries and multicenter or single-center data; however, it should be underlined that many of them are interrelated, specific to the type of the transplanted organ and the age of transplant recipient; therefore, the data are not always consistent. In general, the risk factors for early and late PTLD differ. Pediatric age of recipient (mainly <10 years of age), primary EBV infection, and use of high-dose depleting polyclonal antibody induction have been identified as risk factors of early PTLD, while long-term maintenance immunosuppression and older (adult) age of recipients are indicative of late PTLD. However, there are several confounding factors in these associations, such as cumulative dose and specific type of previously and currently used formulations of biologic drugs (in induction) and variable virulence of specific EBV strains [13]. Seronegative status of EBV before transplantation and donor/recipient EBV mismatch (D+/R-) are regarded as age-independent significant risk factors for further development of PTLD. Analysis of data from Organ Procurement Transplantation Network/United Network for Organ Sharing (OPTN/UNOS), including a total of 137,939 primary kidney recipients (3,907 children and adolescents), showed that adjusted risk (HR_a_) of PTLD in the pediatric subpopulation within 3-year follow-up, associated with (deceased) donor/recipient (D/R) EBV serostatus, is 17.39 in the case of a D+/R- mismatch, compared to the reference (R+). The same parameter was lower in the adult setting (HR_a_ 6.19); however, it was also significantly higher, as compared with the relevant reference (R+). Comparison of the overall risk of PTLD revealed that adjusted risk (HR_a_) in patients <18 years was 2.01 vs. 0.40 for the age of 18–40 years (and deceased donor transplants). These data are consistent with previous reports, indicating patients <10 years of age as the main subgroup of pre-transplant EBV-seronegative status and further risk of NHL. Such patients often receive grafts from older and EBV-seropositive donors, which leads to a relevant mismatch [21–23]. EBV is linked to a variety of lymphomas, including three major B-cell malignancies, Hodgkin and Burkitt lymphoma and NHL DLBCL. Similar types of tumor can also occur in EBV-negative forms; however, the presence in situ of active virus genome cells in a tumor confirms its etiological role in relevant cases. This link is associated with the ability of EBV to transform and immortalize lymphoblastoid B cell lines (LCLs), resulting in proliferation and in secondary genetic and epigenetic mutations, and with its ability to protect them against natural cell apoptosis [13, 24]. EBV also induces the production of latent membrane oncogens (LMPs) and expresses six EBV-associated nuclear antigens (EBNAs), which leads to the proliferation of infected cells. EBV-induced proliferation of cells and specific combinations of EBV variant/HLA types may enable such proteins to evade the immune control [2, 25]. The disturbances of innate and adaptive immune reactions, including EBV-specific CD8 T lymphocyte (CTL) responses, are important for controlling EBV infection; therefore, in EBV-naïve recipients of SOT facing primary EBV infection during the early post-transplant period, the delay in specific anti-EBV immune response caused by T cell-targeted immunosuppression impairs control of the disease [26]. More than 70% of pediatric PTLDs are EBV-positive. CMV seronegativity has a minor effect on PTLD risk, as compared with EBV [23]. Reactivation of EBV infection in EBV-positive (at transplantation) children is variable. It was reported within a range of <20 to 74% [27, 28].

## Impact of immunosuppression on risk of PTLD/NHL

Depleting induction was traditionally regarded as a potentially important risk factor of developing NHL. A report based on the Transplant Cancer Match Study, including 111,857 adult patients after kidney transplantation, revealed that specific biologic agents which significantly increased the incidence of NHL were alemtuzumab (aIRR 1.79) and muorab-CD3 (aIRR 1.37), while the use of polyclonal depletive agents (aIRR 0.96) or anti-IL2R monoclonals (aIRR 0.82) was not associated with a higher risk of NHL. However, it must be noted that this registry data did not include records on EBV status, which (additionally to the adult age of recipients; from 43 to 48 years in subgroups) would be probably a significant co-factor of NHL risk and might potentially modify the effect of polyclonal depleting induction (in cases of EBV mismatch). Some of these associations cannot be currently validated, as muorab-CD3 is no longer used, the formulas (and dosage) of polyclonal agents have been changed over time, and the use of alemtuzumab is limited [29]. The high risk of PCNSL must be considered in patients planned to receive co-stimulation blocker—belatacept, as about 44% of NHL cases found in EBV-seronegative (prior to transplant) adult cases treated with this drug were of CNS location. Pre-transplant seronegative status of EBV, typical in the first decade of life, limits the safe use of this drug in children [13, 30]. In general, long-term maintenance immunosuppression including calcineurin inhibitors and mycophenolate mofetil (MMF) is regarded as a risk factor of late-onset PTLD [15]; however, real impact of every particular drug on this risk is not clear. The increased rates of PTLD beyond the 1990s (after the widespread introduction of tacrolimus to clinical practice) suggested that link; however, the dosing of tacrolimus two decades ago was significantly higher than currently [31]. The widespread introduction of MMF to routine maintenance protocols, in general, did not increase the risk of malignancy. OPTN/UNOS data (adult patients on MMF plus matched controls not receiving MMF) showed a trend towards lower risk of developing lymphoma in patients receiving MMF (0.53% vs. 0.95% in patients not receiving MMF) and no difference in terms of time to develop lymphoma between MMF-treated vs. non-MMF-treated patients. However, it cannot be excluded that reduced exposure to tacrolimus in MMF-treated patients might be reflected in a decreased risk of developing lymphoma [32]. A German pediatric study comparing early-onset vs. late-onset PTLD (B-cell NHL in majority in both settings) showed that tacrolimus and MMF have been used more frequently in patients presenting early than late PTLD (65.8% vs. 31.9%; *p* = 0.001 for TAC and 47.4% vs. 18.8%; *p* = 0.0034 for MMF); however, these data have been collected between 1991 and 2011, and statistical correlations were combined for all SOT recipients (52 kidney, 28 liver, 40 heart, 4 heart/lung, 2 lung, and 1 small bowel transplant recipient) and therefore cannot be directly extrapolated to the subgroup of kidney recipients treated with currently the most common immunosuppressive protocol (TAC/MMF/Pred) [15]. Despite potentially important properties of mTORi against the development of PLTD [33], the data of OPTN/UNOS show that the combination of TAC/mTORi was associated with a slightly higher risk (HR 1.40) of PTLD, compared with TAC+MMF-based protocol [23]; however, this is probably an effect of the net strength of triple immunosuppression. The presence of sirolimus in the quadruple immunosuppressive protocol, also including calcineurin inhibitor, basiliximab, and short-term steroids aimed at steroid minimization, did not prevent the development of PTLD (a high incidence of 6.9%) in young, EBV-seronegative children after kidney transplantation [34]. The data on the association between specific immunosuppressive drugs and PTLD are presented in Table 3, based on data from OPTN/UNOS, including 114,025 kidney transplant recipients, of whom 754 (0.84%) developed PTLD during 5 years after transplantation. These data combine the evaluation of the effect of induction and the commonly used maintenance protocols (not single drugs) and additionally the impact of pre-transplant EBV status, on the risk of PTLD in patients treated with different combinations of immunosuppressive drugs [23].

**Table 3.** Associations between immunosuppression (maintenance drugs as at discharge) and the risk of PTLD [23]

**Immunosuppression**	Adjusted hazard ratio All recipients	Adjusted hazard ratio EBV-positive recipients	Adjusted hazard ratio EBV-negative recipients
**Induction**			
Thymoglobulin	1.34 (*p*<0.01)	1.32	1.31
IL-2RA	0.88	1.03	0.74
**Maintenance** (*TAC+MMF as reference)			
Steroids (at discharge)	1.12	1.27	0.03
CsA+mTORi	0.9	1.12	0.84
TAC+mTORi	1.4 (*p*<0.05)	0.93	1.98 (*p*<0.01)
CsA+MMF	0.8 (*p*<0.01)	1.00	0.45 (*p*<0.01)

*IL-2RA* monoclonal ab. blocking IL2 receptor, *Tac* tacrolimus, *MMF* mycophenolate mofetil, *CsA* cyclosporine, *mTORi* mammalian target of rapamycin inhibitor

## Diagnostic and surveillance approach

The diagnostic approach should cover four timeframes: the time directly before transplantation, the long-term follow-up after transplantation, with a surveillance protocol aiming to monitor selected patients at risk, time point of primary diagnosis of PTLD/NHL, and follow-up of response to relevant therapy. This algorithm may be modified in patients listed for re-transplantation after being cured of PTLD/NHL. Pre-transplant evaluation is mainly aimed at defining compatibility of EBV status between donor and recipient, as a mismatch (D+/R-) is a risk factor. In most cases, serologic tests are performed. Post-transplant follow-up in patients at risk involves regular monitoring of whole blood EBV viral load (EBV-DNA-emia) with the real-time EBV PCR technique. The time intervals between consecutive tests vary (depending on the policy of a center), however should not exceed 3**–**6 months in patients at risk. The data on the value of surveillance protocols are not consistent, as they depend on the specificity of the patient population, the variable pre-transplant status of donor/recipient EBV, age, transplanted organ, degree of reduction of maintenance immunosuppression, and the use of antiviral drugs [17, 35, 36]. The cut-off for positive PCR result for EBV DNA was set as 3,000 copies/μL in an Italian pediatric kidney transplant study, and positive patients were subsequently evaluated for possible development of PTLD with repeated abdominal scan (every 6 months) and chest X-ray (once a year). Reactivation of EBV-positive patients (at transplantation) was below 20% within a mean follow-up of 4.48 months. Median value >59,909 EBV copies was a significant and independent predictor of non-early lesion PTLD and all PTLDs [27]*.* Post-transplant EBV viral load with a threshold of >10,000 copies/mL was predictive for overall survival in patients with NHL (monomorphic diffuse large B-cell lymphoma (M-DLBCL)) receiving relevant treatment, indicating the value of post-transplant monitoring in symptomatic patients [37].

## Clinical monitoring and imaging

Careful physical examination, performed at every outpatient visit, should be in relevant cases followed by:

- A panel of routine blood tests, kidney transplant function evaluation (including urate concentration), blood morphology (screening for leuco-/thrombocytopenia and anemia), lactate dehydrogenase (LDH), liver function, and viral tests [35]
- Imaging tests which may include ultrasonography (including contrast-enhanced USG), computed tomography (CT) scan, magnetic resonance imaging (MRI), and positron emission tomography/computed tomography (PET/CT) of the neck, chest, abdomen, pelvis, or CNS, depending on individual indication [38, 39]

Data from a meta-analysis of reports on PTLD imaging in adult patients showed that the introduction of ^18^F-fluorodeoxyglucose [18F]FDG PET/CT has increased the rate of detection of additional lesions (previously not detected by standard CT and MRI) by 27.8%, from which extranodal sites were involved in 23.6% [40]. Intestinal endoscopy may also be required to evaluate the specific site of a malignant lesion [41, 42].

Imaging is a basic diagnostic tool used to determine the stage of malignancy [16]. Lumbar puncture evaluation is reserved for patients with neurological symptoms and CNS localization of the tumor. Pathological investigation requires needle core biopsy. The range of diagnostic techniques includes major (mandatory) evaluations [35]:

- Morphology (interpretation according to the current WHO classification of PTLD)
- Immunohistology and EBV-encoded RNA in situ hybridization (EBER ISH) and supplementary tests, including:
- Molecular genetic evaluation of antigen receptor genes
- Fluorescent in situ hybridization (FISH)
- Immunoglobulin rearrangement (light/heavy chains)

## Treatment

In general, the basic goal in post-transplant therapy of lymphomas is to cure the disease, however with the protection of allograft function, if possible. The treatment principles include the reconstitution of anti-EBV and/or antitumor immune responses and, if there is no effect, the immuno-/chemo/radiotherapy of malignancy [2]. Before therapeutic decisions on step-wise approach, the final diagnosis must be verified by a core multidisciplinary team, including an experienced radiologist, pathologist, pediatric hemato-oncologist, transplant physician, and surgeon. There are adult guidelines available [17, 43]; however, the overall number of controlled trials related to specific treatment of NHL developed in pediatric patients after kidney transplantation is limited. On the other hand, pediatric patients may present distinct and, in general, more favorable clinical outcomes of post-transplant NHL. The first step of management, used for several years, is a reduction of (exposure to) immunosuppression (RI), aimed at the reconstitution of the immune response. RI may be regarded as pre-emptive management of mild forms of PTLD and as a part of complex treatment of lymphomas. In cases of B-cell-positive NHL, a reduction of exposure to antiproliferative drugs (AZA/MMF) and calcineurin inhibitors (CNI; cyclosporine /tacrolimus) (up to complete withdrawal) should be accompanied by direct administration of anti-B cell monoclonal antibody (rituximab). Exposure to steroids should not be reduced in any situation, and baseline dose may be in some cases increased to values required in oncologic protocols of NHL-related chemotherapy [17]. Nevertheless, data on associations between the risk of PTLD and the use of mTORi are unclear; there are centers where pre-emptive switch from CNI to mTORi is used as a specific form of RI, however with no conclusive results [44]. One of the targets of RI is a reduction of EBV viral load in positive patients. This was reported in an Italian pediatric kidney transplant study, where RI (mainly based on withdrawal of MMF) resulted in reversion of viral load to the values <3,000 copies/μL in 38% of patients [27]. Some patients after kidney transplantation develop a long-lasting chronic high load of EBV despite RI. It recently was reported with an incidence of 24%, starting at a median of 69 days and lasting for a median time of 2.3 years post-transplant. Young age (median age of 2 vs. 12 years; *p* = 0.0001) was associated with this phenomenon. Notably, none of these patients developed PTLD [45]. It must be stressed that a significant conflict of interest exists between the reduction of immunosuppression, aimed to control the development of PTLD (or lymphoma) and the general purpose of organ transplantation, as the former increases the risk of acute and chronic rejection, graft loss, and poorer patient survival. Rejection rate up to 36.8% was reported in a pediatric study due to RI; nevertheless, in the majority of cases, only MMF was stopped [27]. CNI withdrawal is more harmful and was reported as associated with a 3 times higher risk of graft loss (HR = 3.07) [46, 47]. Therefore, after the stratification of the risk/benefit ratio, a careful reduction of CNI exposure should be undertaken [48]. On the other hand, maintaining sufficient kidney graft function in children treated for post-transplant NHL is available and was reported [47, 49], including cases of no significant decrease of eGFR in 3-year follow-up after successful treatment of NHL. In general, the risk of acute rejection and graft loss in children who had their immunosuppression reduced due to NHL seems to be lower than in adult patients [47]. Apparently, management limited to the reduction of immunosuppression (RI) will not be sufficient in more severe cases, including confirmed NHLs. This also raises the question about the optimal duration of the waiting time required to verify who will and who will not respond to RI. Clinical response allows avoiding toxic chemotherapy; however, lack of response confirmed too late may delay the introduction of a relevant therapy. Two approaches described as therapy introduced “sequentially vs. directly after failed RI” have been discussed; however, optimal waiting time was not clearly defined [50]. In adults with a stable early stage of PTLD, a waiting time of 6 weeks to verify the response to IR was suggested. The usual factors of poor prognosis identified in adult patients, including stage III/IV of malignancy, involvement of the allograft, and elevated LDH, should predict a lack of response to RI [51, 52]. In children, poor predictors of response to RI included EBV and B_CD20_ negativity, late-onset PTLD, CNS involvement, and Burkitt or Hodgkin lymphoma morphology [2, 3]. The next step includes B_CD20_ cell depletion by anti-CD20 antibody. Rituximab has been an integral part of B cell NHL therapeutic protocols for years. In the adult population, it was used as a second-line (post-RS) treatment for non-destructive, polymorphic, and monomorphic PTLD (as monotherapy) or in combination with chemotherapy in all non-DLBCL CD20+ monomorphic subtypes, in sequential treatment or risk-stratified treatment (with CHOP), and in prospective clinical trials in adult patients [53]. The success rate in monotherapy in adult patients was variable, with 42% of patients achieving complete response, 17% partial response, and with progressive disease in 41% of patients. The achieved remissions were relatively short-term, and 26% of responders were re-treated within 1 year; however, this improved the rates of complete response in 61% of partial responders [3]. Rituximab in monotherapy induced a significant decrease of EBV load to an undetectable level in EBV-positive pediatric patients after kidney transplantation; however, this effect was time-limited and disappeared within 2–6 months, and viral load returned to baseline in most of the cases. Nevertheless, none of those patients developed malignancy in follow-up (max. 60 months) [27]. Combination (in a sequential manner) of rituximab with chemotherapy used in B_CD20+_ lymphomas in adult SOT recipients has decreased treatment-related mortality, probably due to the additional effect on decreasing tumor mass. Response to rituximab was also a prognostic factor in this study [54], and this predictive parameter was used in another sequential trial (*n* = 152; 45% kidney transplants) prospective study, where patients with sufficient response to four doses of rituximab (verified by repeated CT evaluation of staging) were not receiving further chemotherapy [55]. Another relevant recent adult study has confirmed the suitability of this approach, however indicated the importance of preliminary risk stratification, as in high-risk disease monotherapy with rituximab was associated with a lower complete response rate (21% vs. 68%; *p* = 0.006). The final conclusion of this study was that upfront R-CHOP protocol may benefit individual high-risk cases in whom rapid attainment of response is desirable [56]. In the pediatric population, this approach was used in a large non-randomized prospective multicenter study to test a response-adapted sequential treatment with rituximab +/- chemotherapy in pediatric patients (18 kidney, 11 liver graft recipients, and 20 patients after heart or lung transplantation with CD20+ PTLD without CNS involvement (including 17 patients with early and 32 with late PTLDs; 12 patients with polymorphic histology, 24 with DLBCL, 7 with Burkitt lymphoma, and 6 patients with other high-grade B cell lymphomas upon central review). They were treated with three weekly infusions of rituximab at a dose of 375 mg/m^2^. If at least a partial response was obtained in week 3, patients received three further doses of rituximab every 3 weeks. In case of stable disease or progression, patients were stratified to receive a moderate chemotherapy regimen (mCOMP). Thirty-two patients (64%) received only rituximab, of whom 26 (81% of responders, 53% of total study population) remain alive and in continuous complete remission with a median follow-up of 4.9 years. The remaining 6 rituximab responders experienced relapse (*n* = 4), secondary malignancy (*n* = 1), or death unrelated to PTLD (*n* = 1). Fifteen patients had stable or progressive disease after initial rituximab treatment and proceeded to chemotherapy. Overall, chemotherapy could be spared in about half of PTLD patients [57]. Low-dose or high-dose chemotherapy was used in pediatric PTLD, depending on the setting and severity of the malignancy. Low dose included six 3 weekly courses of cyclophosphamide 600 mg/m^2^ at day 1 together with prednisone 1 mg/kg/day (days 1–5). This protocol eliminated vincristine and daunorubicin from the classic CHOP protocol [58]. A phase II trial adding six doses of rituximab to a low-dose cyclophosphamide and prednisone regimen was conducted for 55 pediatric patients (31% kidney recipients) with EBV+ and CD20+ PTLD (73% presented disseminated III/IV stage disease). The complete remission (CR) rate was 72% in renal patients. There were 10 deaths, 3 due to infections while receiving therapy and 7 from PTLD. The 2-year event-free survival (alive with functioning original allograft and no PTLD) was 64% in renal patients. Due to small numbers, the authors were unable to determine the significance of tumor histology, stage of disease, allograft type, or early response to treatment for outcome [59]. Seven pediatric kidney transplant recipients (among 30 SOT recipients) presenting in majority polymorphic polyclonal B-cell EBER-positive NHL received another protocol, where a combination of rituximab and reduced dose chemotherapy was used directly after failed reduction of immunosuppression with a good result (100% of complete response and 12% of recurrence) [50]. Variable, individual case-adjusted oncologic protocols of chemotherapy (according to R-CHOP, 3 LMB protocol with the addition of rituximab, CHOP followed by COP) were used in the treatment of NHLs after kidney or liver transplantation [49]. Another distinct approach, based on pre-emptive therapy with rituximab of selected pediatric patients after kidney transplantation presenting a high EBV viral load >1,000 copies/mL (median 171,639 copies/mL), was reported by a Korean group. None of such treated patients developed PTLD within a median follow-up of 51.5 months. Apparently, this protocol will not be useful in patients with overt NHL [60]. Active replication of EBV contributes to the pathogenesis of EBV-positive PTLD; therefore, the potential efficacy of antiviral prophylaxis or treatment was the subject of interest. There were published data on its efficacy in kidney and liver transplant recipients in terms of primary infection and increased clearance of EBV [61–63]; however, a recent pediatric report denied any effect of 3-month valganciclovir prophylaxis (otherwise applied as anti-CMV prophylaxis) on the risk of PTLD [27]. Tumor cells latently infected by EBV do not express specific viral protein kinase, crucial for drug activity [2, 3]. It was the basis of a preliminary (adult) study, involving the induction of viral lytic cycle with arginine butyrate (as the inductor) and using ganciclovir in this condition in adult patients (including SOT, 1 kidney) with different types of lymphoma. Drug toxicity was a notable problem [61]. A relevant, more extended clinical trial is planned in the adult population (NCT04337827) [3]. Radiation of localized lesions [64] is a limited option in a pediatric setting, and its use is reduced to field radiation in NHL/Hodgkin-like lymphomas localized in the CNS [65]. Patients who were successfully treated for post-transplant NHL and maintained the graft function received (after gaining control over malignancy) variable long-term immunosuppression, including the use of mTORi or MMF or low-dose CNI with steroids, which allowed acute rejection to be avoided [47].

**Fig. 1.**
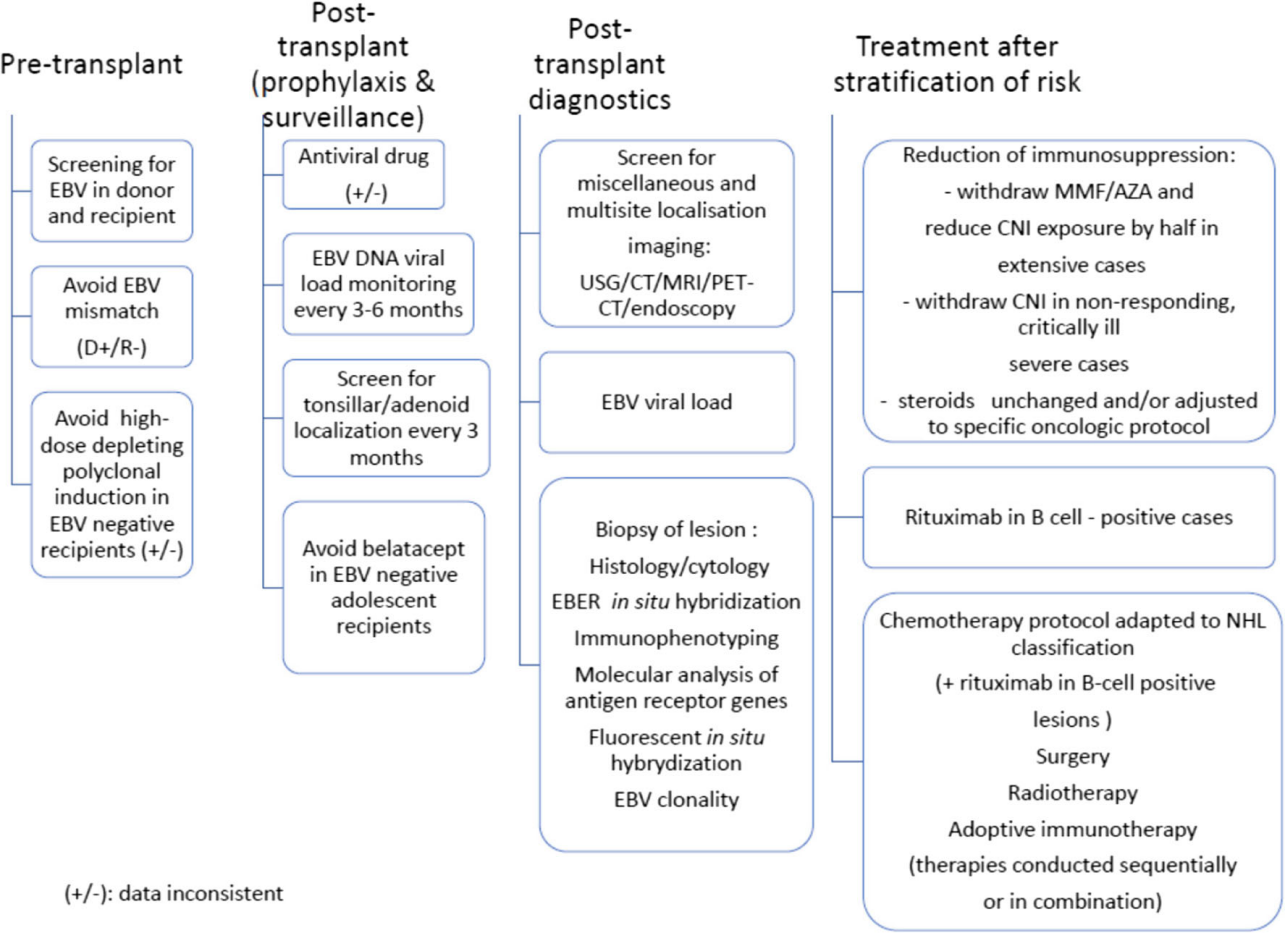
Management of PTLD/NHL in patients after pediatric kidney transplantation at risk (adapted from [13, 35, 38, 43, 53, 66]). EBV, Epstein-Barr virus; NHL, non-Hodgkin lymphoma; MMF, mycophenolate mofetil; AZA, azathioprine; CNI, calcineurin inhibitor; USG, ultrasonography; CT, computed tomography; MRI, magnetic resonance imaging; PET-CT, positron emission tomography/computed tomography; EBER, EBV-encoded RNA hybridization

The summary of therapeutic options is presented in Table 4. The summary of diagnostic and therapeutic approach in PTLD/NHL is presented in Fig.1.

**Table 4.** Therapeutic modalities in PTLD (adapted from [53]; modified in comments).

**Modality of management**	**Mechanism of action**	**Indication**	**Comments**
Reduction of immunosuppression (RI)	Restoration of T-cell function, in particular, EBV-specific T-cell response	Pre-emptive therapy in high-risk patients and first-line management of all types of PTLD	Monotherapy in mild PTLD and part of complex therapy in lymphomas Degree of RI adjusted to severity (stage) of malignancy Clinical and lab-based verification after <2–4 weeks in early/mild PTLD; high LDH suggests resistance to RI (mainly in adult patients) - RI is combined directly with rituximab in more advanced PTLD/NHL RI increases risk of allograft rejection, which is higher in adult patients than in children
B_CD20_ depletion (rituximab)	Reduction of tumoral mass	Second-line (post-RS) treatment for non-destructive, polymorphic, and monomorphic PTLD Combined with chemotherapy in all non-DLBCL B_CD20+_ monomorphic subtypes	Limited to B_CD20+_ types of PTLD Risk of infection
Chemotherapy	Reduction of tumoral mass	Non-destructive, polymorphic PTLD, monomorphic DLBCL in cases mot-responding to IR + rituximab Lymphoma-specific therapy for other (non-DLBCL) monomorphic subtypes	High response rates Risk of infection
Antivirals	Targeting EBV	May be effective in combination with viral thymidine kinase-inducing agents	Limited to EBV-positive cases No efficacy in monotherapy (absence of thymidine kinase expression in EBV-positive PTLD)
Adoptive immunotherapy (EBV-specific cytotoxic T-cells)	Restoration of EBV-specific T-cell response	Relapsing or refractory PTLD	Limited to EBV-positive cases; high costs at limited availability
Radiotherapy	Reduction of tumoral mass	In selected cases: after chemotherapy in HL Whole-brain radiotherapy in PNCSL, if chemotherapy contraindicated	
Surgery	Reduction of tumoral mass	Limited stage of disease Palliative care	Combined with other therapies
High-dose therapy and autologous HSCT	Reduction of tumoral mass	Relapsing or refractory PTLD	Limited experience

## Prognostic factors of outcomes

Several factors associated with a worse outcome of post-transplant PTLD and NHL have been identified. CNS, bone marrow and multi-organ involvement, advanced stage of the disease, involvement of allograft, involvement of >1 extranodal site, and elevated LDH (and older age in adult patients) have been reported as significant factors of poor outcomes and inferior patient survival [52, 66, 67]. The overall survival (OS) rate was proposed as a factor stratifying the overall risk to (1) low (still alive), (2) intermediate-low risk (median OS of 6.8 years), (3) intermediate-high risk (median OS of 1.8 years), and (4) high risk (median OS of 1.3 months) [3, 15]. The combination and cumulative number of specific risk factors may serve as a prognostic index [15].

## Novel therapies and clinical trials

Data on grading of oncologic treatment-related toxicities according to Common Toxicity Criteria for Adverse Events (CTCAE) are present in most relevant reports [68]. They are related to specific drug-related variable adverse events (e.g., related to the use of doxorubicne, vincristine, or cyclophosphamide) or infectious complications in neutropenic patients. Adverse events decrease the tolerability of the whole regimen and are especially important in the transplant setting presenting several comorbidities. This, together with efforts aimed to improve the final efficacy of the treatment, stimulates active research on novel therapies. There are currently nine phase I to III clinical studies in PTLD, mainly recruiting adult patients, aimed at developing new therapeutic strategies in relapsed/refractory or untreated malignancies. One of those (NCT03392142) recruits pediatric patients with refractory EBV+ PTLD and is focused on tabelecleucel in patients presenting a poor response to rituximab consolidation. Tabelecleucel comes from T cells collected from the blood of third-party donors, exposed to EBV antigens and managed for future therapeutic use (as adoptive T cell therapy) [3]. Other novel therapies, such as the combination of rituximab with brentuximab vedotin (immunoconjugate), with EBV cytotoxic T cell therapy (CTL) (for CD30+ EBV+ post-transplant lymphomas), and acalabrutinib (oral inhibitor of Bruton’s tyrosine kinase) have not been investigated in the pediatric transplant population [3]. The promising results of the off-the-shelf EBV-specific T cell immunotherapy for rituximab-refractory EBV-associated lymphoma following SOT have been recently reported [69]. A specific system for the rapid generation of EBV-CTLs resistant to tacrolimus was developed, based on the selection of interferon-gamma-secreting EBV-CTLs and retroviral transduction with calcineurin B mutant cells [70]. This protocol allows production of cytotoxic T lymphocytes resistant to the basic immunosuppressive drug and potentially suitable to treat PTLD/NHL [3].

## Re-transplantation after cured NHL

Long-term patient survival after primary kidney transplantation and increasing efficacy in treating lymphomas are reasons for re-transplantation in cases with a history of malignancy. The majority of reported cases are adult patients; however, pediatric cases have also been described, transplanted primarily as children and then re-transplanted as adult recipients after several years. A series of 8 patients, including 5 children aged 1–11 years at primary kidney transplantation, who developed lymphomas of variable localizations (3 B-cell NHLs, one B+T cell, and one Burkitt lymphoma) within a wide range of time from 5 months to 24 years after kidney transplantation and were treated with chemotherapy (or R-CHOP), was reported by a Minnesota Center research team. All patients lost their grafts (mainly to chronic rejection) and received subsequent transplants at 51 to 95 months after PTLD, with no relapse of malignancy; nevertheless, 4 of them received polyclonal induction at the second transplantation procedure. Developing immunity against EBV (seroconversion) before re-transplantation was underlined as an important factor of success. Authors discussed the optimal (minimal) duration of waiting time of malignancy remission before qualification to subsequent transplantation and suggested that a PTLD-free period of at least two to three years after remission is reasonable, accompanied with sustained remission of EBV viremia and gaining seropositive EBV status in previously seronegative patients [71]. A period of at least 1 year from the control of PTLD to re-transplantation (in adult patients) was suggested in the guidelines of the British Transplantation Society [35]. Data from OPTN/UNOS database included 27 cases of kidney re-transplantation (39.1% of all 69 SOTs) in patients with a history of malignancy. Twelve renal patients (44% of all kidney recipients) were under 18 years of age at primary transplantation. The median time to develop PTLD was 1042 days in renal patients. The major cause of further kidney graft loss was chronic or acute rejection due to the reduction of immunosuppression. The median time from PTLD to kidney re-transplantation was 1337 days. All 27 patients remained alive and 24 (88.9%) re-transplanted kidneys were functioning at a mean follow-up of 742±107 days [72]. Data from the French PTLD Registry included 52 patients undergoing 55 kidney re-transplantations after PTLD developed following primary transplantation. Four of them (8%) were pediatric cases at first transplantation. The majority of PTLDs (67%) were EBV-positive and had monomorphic (78%) lesions. The time interval between PTLD and re-transplantation was 100±224 months. All but one developed immunity against EBV before subsequent transplantation. A total of 31 patients received depleting polyclonal induction, and 57% received blocking induction (IL-2R antagonist), and 6 received rituximab and 53% an antiviral drug. All received triple maintenance immunosuppression. None of them (except one) experienced a recurrence of PTLD [73].

### Key summary points

- Non-Hodgkin lymphoma (NHL) developing after kidney transplantation belongs to the group of post-transplant lymphoproliferative disorders (PTLD) and in specific series may represent up to two-thirds of cases of PTLD
- The risk of developing NHL in pediatric solid organ recipients is about 200 times higher than in the age-matched general pediatric population; therefore, more than half of post-transplant malignancies are lymphomas.
- The majority (about 65%) of the confirmed cases of NHL are diffuse large B-cell lymphomas (DLBCL).
- Risk factors of developing NHL are in general identical as those for PTLD: long-term maintenance, as well as some forms of initial depleting immunosuppression, seronegative EBV status before transplantation in young recipients, and donor/recipient mismatch of EBV.
- Basic goal in NHL therapy is to cure the disease with maintaining allograft function, which may not be easily achievable due to an increased risk of rejection, entailed by reduction of immunosuppression; however, this risk is lower in children than in adult patients.
- Step-wise management includes reduction of maintenance immunosuppression (all patients) and use of B_CD20_-cell depletion (in CD20-positive cases), followed by chemotherapy in non-responding and more severe cases of NHL.
- Final outcome depends on staging/subtype of malignancy and individual tolerance of toxic oncologic treatment; however, high success rates of survival have been reported in several cases treated with reduction of immunosuppression and rituximab.
- Stage IV of progressive disease, especially of CNS (central nervous system) localization, is related to worse prognosis.
- Successful kidney re-transplantation was reported in cured patients after careful verification of malignancy-free status, gaining adaptive immune response to EBV and about >2–3 years of waiting time.

### Multiple-choice questions

1. Non-Hodgkin lymphoma (NHL) in patients after kidney transplantation:

- Is a unique and very rare malignancy, seen mainly in adults
- Is a common type of PTLD
- Develops only >10 years after transplantation
- Presents incidence comparable with normal age-matched population
- Is diagnosed only in adolescents after transplantation

2. Non-Hodgkin lymphoma (NHL) after kidney transplantation:

- Is EBV-negative in all cases
- Never develops in CMV-negative patients
- Develops only in EBV-seronegative patients before transplantation
- Is EBV-positive in the majority of pediatric patients
- Never develops in patients presenting EBV reactivation

3. Confirmed associations between immunosuppression and risk of developing NHL include:

- Importance of high exposure to steroids, which must be withdrawn after diagnosis
- Use of anti-IL2R-based blocking induction
- Use of rituximab in the treatment of primary nephrotic syndrome in case-history before transplantation
- Use of belatacept in EBV-negative (at transplant) patients
- Use of moderate doses of rabbit thymoglobulin for induction in EBV-positive patients

4. Therapeutic approach in post-transplant NHL includes:

- Immediate radiation of the malignant lesion, regardless of its localization
- Use of rituximab monotherapy in all cases
- Immediate use of interferon together with rituximab
- Reduction of immunosuppression plus rituximab in B cell-positive cases and specific oncologic protocol, adjusted to the defined staging of the disease and morphology of the malignant lesion
- Intensive antiviral treatment (ganciclovir for 6 months) and with IVIG pulses (2 g/kg) in EBV-positive cases, combined with rituximab

5. In patients who survived NHL and lost the kidney graft:

- Re-transplantation is not possible due to unacceptable, high risk of malignancy recurrence
- Re-transplantation must be postponed >10 years after malignancy
- Immunosuppression in re-transplantation must be reduced and never include biological agents
- Re-transplantation is possible after 2–3 years of malignancy-free period and after gaining immunity against EBV (in previously negative patients)
- Re-transplantation must be performed with prophylactic use of rituximab
